# Age Associated Microbiome and Microbial Metabolites Modulation and Its Association With Systemic Inflammation in a Rhesus Macaque Model

**DOI:** 10.3389/fimmu.2021.748397

**Published:** 2021-10-19

**Authors:** Suresh Pallikkuth, Roberto Mendez, Kyle Russell, Tirupataiah Sirupangi, Daniel Kvistad, Rajendra Pahwa, Francois Villinger, Santanu Banerjee, Savita Pahwa

**Affiliations:** ^1^ Department of Microbiology and Immunology, University of Miami Miller School of Medicine, Miami, FL, United States; ^2^ Department of Surgery, University of Miami Miller School of Medicine, Miami, FL, United States; ^3^ New Iberia Research Center, University of Louisiana at Lafayette, New Iberia, LA, United States; ^4^ Miami Integrative Metabolomics Research Center (MIMRC), University of Miami Miller School of Medicine, Miami, FL, United States; ^5^ Center for Scientific Review, National Institute of Health, Bethesda, MD, United States

**Keywords:** age and microbes, age and metabolites, microbiome and metabolites, immunity and microbiome, age and immunity

## Abstract

Aging is associated with declining immunity and inflammation as well as alterations in the gut microbiome with a decrease of beneficial microbes and increase in pathogenic ones. The aim of this study was to investigate the age associated gut microbiome in relation to immunologic and metabolic profile in a non-human primate (NHP) model. 12 geriatric (age 19-24 years) and 4 young adult (age 3-4 years) Rhesus macaques were included in this study. Immune cell subsets were characterized in peripheral blood mononuclear cells (PBMC) by flow cytometry and plasma cytokines levels were determined by bead based multiplex cytokine analysis. Stool samples were collected by ileal loop and investigated for microbiome analysis by shotgun metagenomics. Serum, gut microbial lysate, and microbe-free fecal extract were subjected to metabolomic analysis by mass-spectrometry. Our results showed that the gut microbiome in geriatric animals had higher abundance of Archaeal and Proteobacterial species and lower Firmicutes than the young adults. Highly abundant microbes in the geriatric animals showed a direct association with plasma biomarkers of inflammation and immune activation such as neopterin, CRP, TNF, IL-2, IL-6, IL-8 and IFN-γ. Significant enrichment of metabolites that contribute to inflammatory and cytotoxic pathways was observed in serum and feces of geriatric animals compared to the young adults. We conclude that aging NHP undergo immunosenescence and age associated alterations in the gut microbiome that has a distinct metabolic profile. Aging NHP can serve as a model for investigating the relationship of the gut microbiome to particular age-associated comorbidities and for strategies aimed at modulating the microbiome.

## Introduction

The world’s population is aging with almost all countries experiencing growth in numbers of old persons. In 2010, an estimated 524 million people were 65 years or older (8% of the world’s population) and the numbers of this age group is expected to grow to 1.5 billion (16% of the world’s population) by 2050. One of the complications of aging is immunosenescence, a series of age-related changes that adversely affect the immune system resulting in increased vulnerability to infectious diseases and comorbidities (1–3). It is not fully understood how the age-related biological changes affect the different components of the immune system, and there is no clear understanding of which changes are primary, arising as a consequence of aging, and which might be secondary, adaptive or compensatory to the primary changes (4). Gaining knowledge of these mechanisms and interactions with multiple components of immune homeostasis will be critical for strategies aimed at delaying the age-related decrease in immunity or preventing its consequences (5–9).

Changes in the immune system that occur with aging are well documented (5, 7, 8, 10). Studies have reported immune alterations such as decrease in antibody response to vaccination (5, 11), increase in inflammatory cytokines in circulation (5, 12, 13) and increased gut permeability, or “leaky” gut (6, 14, 15) associated with aging. We have recently shown that rhesus macaques (RM) represent a good non-human primate (NHP) model for age-associated immune changes (16). As with humans, our aging RM show a decrease in thymic activity that results in less naïve cells being produced, lowering their proportion in both blood and secondary lymphoid organs (17). Concomitantly, there is a relative increase in various populations of memory T cells, in particular, effector memory T cells (17).

An area of increasing interest is the impact of gut microbiome in regulating immune homeostasis. The collection of microbes that exist in the gut of the host are known to undergo changes with age (6, 18–20). Early in adulthood, the microbiome is made up mainly by beneficial microbes from the Firmicutes and Bacteroides phyla (21). With advancing age, the relative abundance of these phyla decrease as the microbiome becomes dominated by more pathogenic microbes from the Proteobacteria phylum leading to microbial dysbiosis (6, 22–26). Age-associated inflammation is associated with microbial dysbiosis through a positive feedback loop whereby microbial products “leak” into the blood stream and increase inflammation (6, 22–24, 27, 28) sustaining the feedback loop (6, 22–24, 27, 28). In the present study we have identified features of inflammation and dysbiosis in aging RM and demonstrated a relationship between age associated microbial dysbiosis, the microbiome and the metabolic state of the host.

## Materials and Methods

### Animals

12 Indian Rhesus Macaques (RM), consisting of 8 geriatric animals (age 19-24 years) and 4 young adult animals (age 3-4 years) were included in this study. Animals were housed and blood/tissue/stool samples were collected at the New Iberia Research Center (NIRC) at the University of Louisiana at Lafayette. Detailed characteristics of animals used including birth date, gender, relatedness and housing information are presented in the **Supplementary Table 1**. All animals used in this study are specific pathogen free (free of STLV, SIV, SRV, Herpes B, and Foamy virus) except for RRV and RhCMV.

### Ethics Statement

All animal experiments were conducted following guidelines established by the Animal Welfare Act and the NIH for housing and care of laboratory animals and performed in accordance with institutional regulations after review and approval by the Institutional Animal Care and Usage Committees (IACUC, # 2016-8798-055) of the University of Louisiana at Lafayette. All efforts were made to minimize suffering and stress. Appropriate procedures were performed to ensure that potential distress, pain, discomfort, and/or injury were limited to that unavoidable in the conduct of the research plan. Ketamine (10 mg/kg) and/or telazol (4 mg/kg) were used for collection of blood and stool samples, and analgesics were used when determined appropriate by veterinary medical staff. This study was carried out in compliance with the ARRIVE guidelines (https://arriveguidelines.org/resources/author-checklists).

### Analysis of Plasma Markers of Immune Activation and Inflammation

Plasma biomarkers of immune activation and inflammation were determined using either an ultra-sensitive, multiplexed Milliplex magnetic bead-based assay panel (EMD Millipore) acquired on a MAGPIX instrument (Luminex Corporation) or commercially available. Multiplex assay was used in the measurement of plasma levels of IL-2, IL-6, IL-8, IL-10, IFN-γ, and TNF-α. Commercial ELISA kits were used in the measurement of plasma levels of, hsCRP (Life Diagnostics), and Neopterin (IBL international).

### Phenotypic Analysis of Immune Cells Subsets by Flow Cytometry

PBMCs were thawed and rested overnight and stained for markers specific for the identification of CD4 and CD8 T cell subsets using Rhesus specific Abs against CD3, CD4, CD8. CD28, CD95 and CCR7 along with live dead aqua dye and acquired on BD Fortessa flow cytometer and analyzed using FlowJo software.

### Gut Microbiome Analysis

Fresh fecal samples were collected from anesthetized animals by insertion of a fecal loop into the rectum and swiping several times and stored at -80°C. The stool samples from both the study groups were collected at the same time in July 2017 and microbiome sequencing was performed for all the samples together. Microbial DNA was isolated from fecal samples using DNeasy Powersoil Kit (Qiagen) and quantified using Qubit DNA analyzer (Invitrogen/Life technologies). DNA samples were sent to the University of Minnesota Genomic Center for whole genome sequencing of the microbial profile. MetAMOS or PartekFlow softwares were used for microbial profiling.

Shotgun metagenomics library was constructed with the Nextera DNA sample preparation kit (Illumina, San Diego, CA), as per manufacturer’s specification. A 94 dual-indexed Nextera XT libraries were prepared for this study. All libraries combined into a single pool and sequenced in a single lane of a HiSeq 2500 HO, 125-bp PE sequencing run using v4 chemistry.Generated ≥ 220 M reads for the lane (an average of 3.5-4 million reads per sample). Mean quality score for all high yield libraries is ≥Q30. Barcoding indices were inserted using Nextera indexing kit (Illumina). Products were purified using Agencourt AMpure XP kit (Beckman Coulter, Brea, CA) and pooled for sequencing. Samples were sequenced using MiSeq reagent kit V2 (Illumina).

Raw sequences were sorted using assigned barcodes and cleaned up before analysis. For assembly and annotation of sequences, MetAMOS pipeline or Partek Flow software (Partek Flow Partek Inc., St. Louis, MO) were used. Alpha and Beta diversity calculations were done using embedded programs within the metagenomics pipeline, or using Stata15 (StataCorp LLC, College Station, TX) or EXPLICET software. Functional profiling was performed using HUMAnN2-0.11.1 (29) with Uniref50 database to implement KEGG orthologies. Sequence files for the samples in this manuscript have been submitted to ArrayExpress with the accession number E-MTAB-10160.

### Dimensionality Reduction and Bacterial Association Analysis

We utilized bacterial-matrix based dimensionality reduction and clustering algorithms. Compared to Qiime-derived PCA, which is done sample-wise, these algorithms are identity agnostic and decipher qualitative association (and disassociations) between experimental groups. We used Graphia software (Edinburgh, UK) and its implementation of Markov Clustering algorithm (MCL) (30). Using our group-wise bacterial matrix, MCL looked for cluster structures using mathematical bootstrapping. While this method is agnostic to phylogenetic hierarchy in the matrix, we used the hierarchy as identifying markers to understand the bacterial clusters and changes in those clusters within the 2 study groups. MCL used the stochastic flow of the matrix to decipher the distances between the bacteria at equilibrium, thereby generating a cluster map by using correlation scores as distance. For generating the cluster, nodes scoring above a Pearson correlation value of 0.85 were used.

### Metabolite Analysis by Mass-Spectrometry

Metabolites from stool and plasma fractions were extracted using chloroform, methanol and water mixture to obtain phase separation. The extracted lipids corresponding to 100 μg proteins were used for IROA (described below) and untargeted liquid chromatography Q-Exactive Orbitrap tandem mass spectrometry (LCMS/MS) for profiling. All solvents were LC-MS/MS grade. Methanol (6 ml) and chloroform (3 ml) were added to each sample (corresponding to 100 μg of total protein from each fraction). After 2 min vigorous vortexing and 2 min sonication in an ultrasonic bath, the samples were incubated at 48°C overnight (in borosilicate glass vials, PTFE-lined caps). The following day, 3 ml of water (LC-MS grade) and 1.5 ml of chloroform were added, samples were vigorously vortexed for 2 min and centrifuged at 3000 RCF, 4°C for 15 min to obtain phase separation. Lower phases were collected. Remaining samples (upper and interphases) were reextracted by addition of 4.5 mL of chloroform and centrifugation at 3000 RCF, 4°C for 15 min to obtain phase separation. Lower phases from both extractions were combined and dried in a centrifugal vacuum concentrator. Samples were stored at -20°C and were reconstituted in 150μL of chloroform:methanol (1:1) before mass spectrometric analysis. To determine the impact of the microbiome on host metabolism in our RM model, we used mass-spectrometry based Isotope Ratio Outlier Analysis (IROA) from RM plasma and feces. This pipeline encompasses sample preparation, LC-MS based peak acquisition, proprietary software-based library creation, normalization and quantification of metabolites and we have standardized and validated IROA method in our laboratory (31). For LC-MS/MS, The Q Exactive (Thermo) mass spectrometer was operated under heated electrospray ionization (HESI) in positive and negative mode separately. The spray voltage was 4.4 kV, the heated capillary was held at 310°C (negative mode) or 350°C (positive mode) and heater at 275°C (positive mode). The S-lens radio frequency (RF) level was 70. The sheath gas flow rate was 30 (negative mode) or 45 units (positive mode), and auxiliary gas was 14 (negative mode) or 15 units (positive mode). Full scan used resolution 70,000 with automatic gain control (AGC) target of 1x106 ions and maximum ion injection time (IT) of 100 ms. MS/MS spectra were acquired using the following parameters: resolution 17,500; AGC 1x105; maximum IT 75 ms; 1.3 m/z isolation window; underfill ratio 0.1%; intensity threshold 1x103; dynamic exclusion time 3 s. Normalized collision energy (NCE) settings were: 15, 30, 45, 60, 75, 90 (in positive and negative mode separately; total 12 runs per sample). Spectral file analysis was done with MZmine2 with integrated Metacyc database. Pathway and disease mapping was performed using MetaboAnalyst 4.0 at https://www.metaboanalyst.ca/MetaboAnalyst/home.xhtml. Metacyc analysis classifies the involved pathways as biosynthesis and degradation pathways, which are essentially anabolic and catabolic pathways respectively.

### Statistical Analysis

A Mann-Whitney U test was conducted to determine whether there was a difference in the plasma cytokine and immune cell phenotypes of the young adult and geriatric groups. The Benjamini-Hochberg correction for multiple comparisons was implemented to calculate adjusted p-values. The alpha level of .05 was used to determine significance. Bray-Curtis dissimilarity matrix was statistically evaluated using PERMANOVA test with Monte-Carlo permutation (999 tests) and Bonferroni correction for FDR. For correlation analysis, microbial data was reordered according to difference in prevalence between young adult and geriatric animals of a given microbe in the microbiome. The 25 microbes most prevalent in geriatric or young adult animals were then correlated with plasma cytokines using prism software. The list of bacteria and cytokines were derived from a Benjamini-Hochberg FDR corrected list. In order to perform the selection, we lined up the list of taxa to look for significantly changing species in geriatric individuals, compared to young adults and vice-versa (with appropriate FDR correction), we picked taxa which had q<0.01 or below. For one comparison, we had 25 taxa that fit the criteria and we picked the top 25 from the other comparison as well. Correlation analyses were performed using Spearman rank order correlation using prism software. Test of significance for this analysis was a simple 2-tailed t-test for the correlation. Heat maps were generated to show the results and significant correlations. Results with a p value <0.05 were considered significant.

## Results

### Higher Systemic Inflammation and Immune Activation in Geriatric Rhesus Macaques

**Figure 1:**
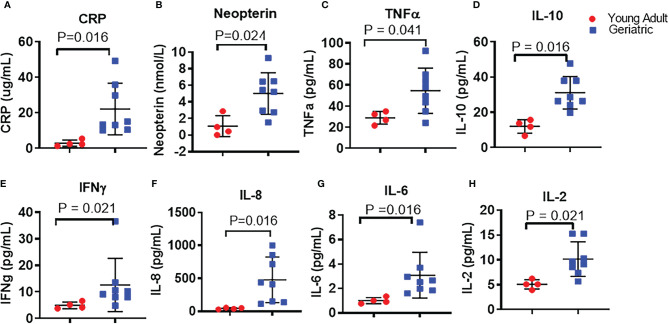
Plasma inflammatory cytokines in young adult and geriatric Rhesus Macaques: ELISA kits were used to quantify levels of CRP **(A)**, and neopterin **(B)** in young adult and geriatric animals. Magpix multiplex analysis was used to quantify levels of TNF **(C)**, IL-10 **(D)**, IFN-γ **(E)**, IL-8 **(F)**, IL-6 **(G)**, and IL-2 **(H)** in young adult and geriatric animals. Statistical analysis performed by Mann-Whitney U test with Benjamini-Hochberg correction for multiple comparisons to calculate the adjusted p-values (q value). Line and whiskers indicate the mean ± standard deviation. Q values are exact and a q < 0.05 was considered significant.

12 RM with 8 geriatric and 4 young adult animals were investigated to elucidate the effects of age on the immune system and microbiome. Analysis of plasma biomarkers depicted in **Figure 1** showed significantly higher levels of acute inflammatory protein CRP (**Figure 1A**), neopterin, a systemic marker of immune activation (**Figure 1B**), and various inflammatory cytokines including TNF (**Figure 1C**), IL-10 (**Figure 1D**), IFN-γ (**Figure 1E**), IL-8 (**Figure 1F**), IL-6 (**Figure 1G**), and IL-2 (**Figure 1H**) in geriatric animals. Analysis of absolute numbers and frequencies of circulating immune cell subsets (**Supplementary Figure 1**) did not show statistically significant differences between groups. However, geriatric animals exhibited a non-significant trend of lower mean absolute numbers of lymphocytes (**Supplementary Figure S1A**) and non-significant trend of higher mean absolute monocytes (**Supplementary Figure S1B**) compared to young adult animals. Within the lymphocytes, geriatric animals showed a non-significant trend of lower mean absolute number of CD4 T cells (**Supplementary Figure S1C**) while mean absolute number of CD8 T cells (**Supplementary Figure S1D**) were similar between young adult and geriatric animals.

In agreement with the trend of lower numbers of CD4 T cells, we also found a non-significant trend of lower mean frequencies of CD4 T cells (**Supplementary Figure S1E**) in geriatric animals. CD4 T cell maturation subsets showed a non-significant trend for lower mean frequencies of naïve (defined as CD4^+^CD28^+^CD95^-^) (**Supplementary Figure S1F**) along with a non-significant trend of higher mean frequency of central memory (defined as CD4^+^CD28^+^CD95^+^) CD4 T cells (**Supplementary Figure S1G**) in geriatric animals. CD4 effector memory (defined as CD4^+^CD28^-^CD95^+^) subset (**Supplementary Figure S1H**) did not differ between the study groups. Frequencies of CD8 T cells (**Supplementary Figure S1I**) did not differ between the groups with the geriatric animals exhibiting a non-significant trend of lower mean frequencies of naïve (**Supplementary Figure S1J**) and central memory (**Supplementary Figure S1K**) and higher mean frequencies of effector memory (**Supplementary Figure S1L** **)** cells. Taken together, our data support an age associated increase in inflammation and a trend for alterations in immune cell subsets in aging NHP that is similar to humans.

### Gut Microbiome Shows Significant Differences Between Young Adult and Geriatric Animals

**Figure 2:**
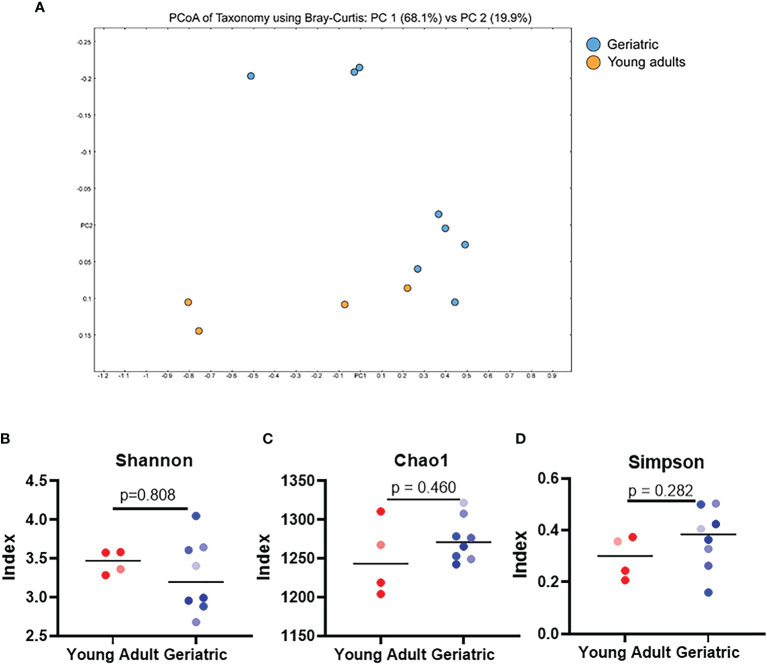
Gut microbial diversity indices of geriatric and young adult animals: **(A)**, Qualitative differences between the two age groups (β-diversity), 2D rendering of the Bray-Curtis dissimilarity matrix showing distinct clustering between the two. **(B–D)** α-diversity indices, namely Shannon **(B)** Chao1 **(C)** and Simpson **(D)** do not exhibit any significant differences between geriatric (blue dots) and young adult (red dots) animals. Color gradients of the dots indicate the age range with lighter color indicate youngest and darker color indicates the oldest animals of respective group. Test of significance **(B–D)** by Mann-Whitney U test, using GraphPad Prism. P-values shown within the graphs are exact.

**Figure 3:**
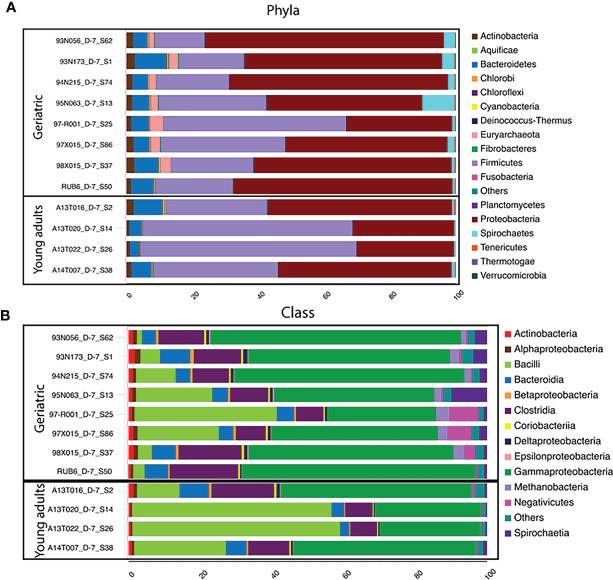
Phylum and Class-level differences in microbiota compositions with age: Stacked bar-plot representation of the percent distribution of microbiota compositions for each animals with taxonomic features at the level of phyla **(A)**, and Class **(B)**. The phyla, and class with low relative abundance are grouped as other. At the phylum level, Rhesus gut microbiome is Proteobacteria and Firmicutes dominated, followed by phylum Bacteroidetes. At the class level, classes belonging to Proteobacteria (Gammaproteobacteria) and Firmicutes (Bacilli, negetivicutes) dominates the landscape.

Stool samples from young adult and geriatric rhesus macaques were analyzed by shotgun metagenomics sequencing (**Figures 2**, **3**). Group-wise compositional differences were determined by Principal coordinate analysis (PCA) using the Bray-Curtis dissimilarity matrix for β -diversity. PCA analysis showed a clear difference in microbiome composition between young adult and geriatric animals without any overlap as shown in the 2D rendering of the Bray-Curtis dissimilarity matrix ((**Figure 2A**). These differences were further visible in the Euclidean and Jansen-Shannon PCA matrices (**Supplementary Figures 2A, B**). While there was a distinct clustering of the young macaque microbiota, the differences were not significant by the PERMANOVA analysis with FDR correction (‘q’ value of 0.57). Diversity changes between the two groups (α -diversity) using three different indices also showed a trend towards age associated decrease in the Shannon index (**Figure 2B**) and increases in Chao1 (**Figure 2C**) and Simpson (**Figure 2D**) indices. An unsupervised hierarchical clustering plot representing each animal is shown in **Figure S2C**. In the young group, 3 animals clustered well while in the old group, 7 animals clustered together.

Investigation of the bacterial phyla and classes showed that the gut microbiome in the animals was dominated by Proteobacteria and Firmicutes (**Figure 3**), followed by phylum Bacteroidetes. Young adult animals showed a Firmicutes dominated microbiota while Proteobacteria dominated in geriatric animals together with relative expansion of Bacteroidetes, Tenericutes and Verrucomicrobia (**Figure 3A**). Despite a decrease with age, Firmicutes still remained the second largest phylum in the microbiome of geriatric animals. Classes within Proteobacteria and Firmicutes consisted of Bacilli (Firmicutes) dominating in young adult animals, followed closely by Gammaproteobacteria (Proteobacteria), Clostridia (Firmicutes), and Bacteroidia (Bacteroidetes). In contrast, in geriatric animals, the most dominant microbiome class was Gammaproteobacteria with greatly diminished representation from Bacilli which constituted the second most abundant class (**Figure 3B**). Clostridia and Bacteroidia occupied the third and fourth most dominant position within the geriatric group and their relative abundance was unchanged compared to young adult animals.

**Figure 4:**
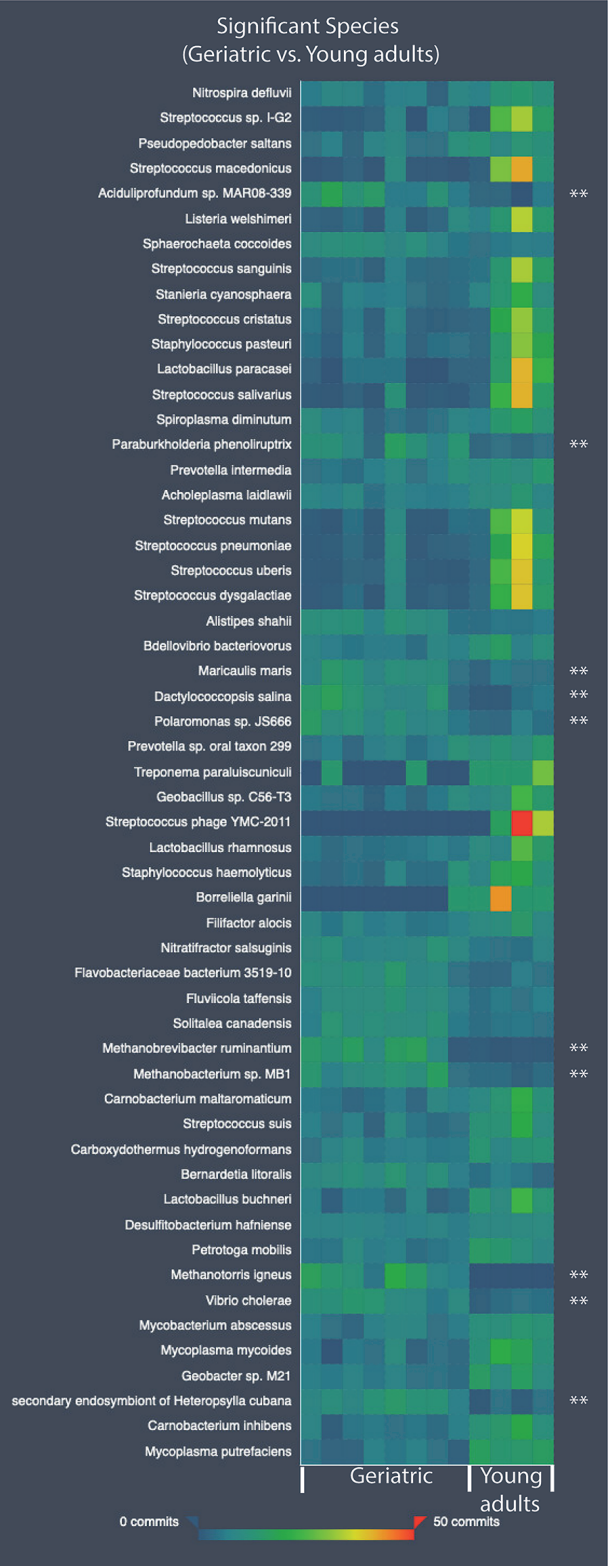
Heatmap showing the relative abundance values for the bacterial species-level differences in the old and young geriatric and young adult animals. All species have significant differences between groups. Species significantly higher in old geriatric animals are marked with ‘**’. The commits in the heatmap are the relative abundance values. Test of significance was pairwise Mann-Whitney U test and p < 0.05 was considered significant.

At the species level, 55 species were significantly different in the gut microbiome of the young adult and geriatric animals (**Figure 4**). As expected, most of the species showing diminished relative abundance in geriatric animals belonged to phylum Firmicutes, while members of phylum Proteobacteria exhibited elevated relative abundance. It was clear from the statistical analysis that there were subtle compositional changes in the microbiome between young adult and geriatric macaques with some significant genus-level changes. While the microbiome changes were modest, we set out to understand the overall physiological role of these changes.

### Relationship of Systemic Inflammation and Gut Microbiome in Geriatric RM

**Figure 5:**
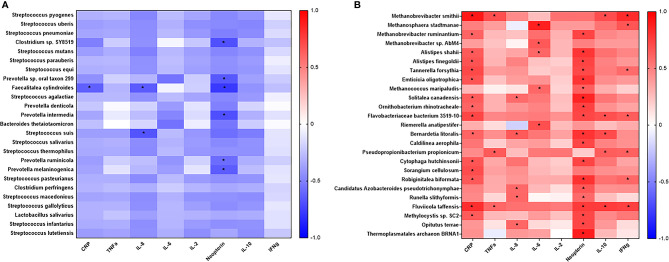
Correlation analysis of prevalent microbes in young adult or geriatric Rhesus Macaques and plasma inflammatory cytokines: Microbes were selected based off increased prevalence in young adult **(A)** or geriatric **(B)** rhesus macaques and then correlated with plasma cytokines. Positive correlations are in red. Negative correlations are in blue. Statistical differences are identified by *. Geriatric animals showed significantly more correlations with plasma cytokines, all of which were positive correlations. The list of bacteria and cytokines were derived from a Benjamini-Hochberg FDR corrected list. Test of significance for this analysis was a simple 2-tailed t-test for the correlation.

To better understand the association between the microbiome and inflammation, a correlation analysis between specific microbial species and plasma inflammatory cytokines was performed. The list of over 600 microbes identified was arranged according to difference in relative abundance between young adult and geriatric animals. Twenty-five microbes representing most abundant microbes in young adult or geriatric animals respectively, were correlated with plasma cytokines (**Figure 5**). In geriatric animals almost all the microbes correlated positively with plasma inflammatory cytokines, with many microbes showing significant correlations with neopterin, CRP, TNF, IL-2, IL-6, IL-8 and IFN-γ. In young adult animals a trend for negative correlation of microbes was noted with the same cytokines, with neopterin showing a significant negative correlation.

Next, we performed correlation modeling of 100 significantly changing microbial species between the two groups and the cytokine levels in young adult and geriatric macaques. For this, we used a Spearman r correlation matrix (presuming a non-gaussian distribution) with 95% confidence interval. Since we had already selected correlates that were corrected for FDR, we performed a simple two-tailed Mann-Whitney U test for significance for the correlation matrix. As shown in **Table 1**, many bacterial species were strongly correlated, positively (red) or negatively (blue) with more than one cytokine in geriatric animals. Interestingly, a majority of these bacterial species were from the phylum Firmicutes that are highly metabolically active and proinflammatory for the host. **Supplementary Figure 3** shows representative matrices for CRP, IL6 and Neopterin and **Table 1** shows the significant correlations with cytokines and bacterial species in the geriatric animals. The correlation matrix showed a recurring theme for cytokines CRP, IL6 and Neopterin, where increased levels correlated positively with various bacterial species in geriatric macaques (seen as warmer palette), whereas in young adults, less correlation was seen for the same cytokines (**Figure 5**). **Table 1** further shows the significant correlations with cytokines and bacterial species in old animals.

**Table 1:** Correlation modeling of plasma cytokine levels with 100 significantly changing microbial species in geriatric animals compared to young adult.

Species	Spearman r	P-value	Cytokine
*Streptococcus equi*	0.7665	0.0323	CRP
	0.7785	0.0295	IL6
	0.7758	0.0292	IL10
*Streptococcus* sp. I-P16	0.8073	0.0208	CRP
	0.7470	0.0411	IL6
	0.7927	0.0246	IL10
*Filifactor alocis*	0.7904	0.0251	IFN-γ
	0.8110	0.0072	IL6
*Staphylococcus pasteuri*	0.7805	0.0292	IFN-γ
	0.9456	0.0016	IL6
	0.7914	0.0270	IL10
*Streptococcus pneumoniae*	0.7425	0.0422	IFN-γ
	0.8333	0.0154	IL6
	0.9157	0.0036	IL10
*Exiguobacterium* sp. AT1b	0.7952	0.0236	IFN-γ
	0.7665	0.0325	IL6
*Streptococcus parasanguinis*	0.7545	0.0382	IFN-γ
*Streptococcus agalactiae*	0.9334	0.0020	IFN-γ
	0.9639	0.0008	IL6
	0.8415	0.0133	IL10
*Streptococcus mutans*	0.7306	0.0485	IL2
	0.7545	0.0382	IFN-γ
	0.8095	0.0218	IL6
*Dactylococcopsis salina*	-0.8072	0.0205	IL2
	-0.9461	0.0011	TNFa
	0.8434	0.0131	IL6
*Natrinema pellirubrum*	0.8729	0.0286	IL6
	0.8835	0.0286	IL10
*Streptococcus salivarius*	0.8333	0.0154	IL6
	0.7591	0.0355	IL10
*Enterococcus faecium*	0.8095	0.0218	IL6
	0.8073	0.0216	IL10
*Listeria welshimeri*	0.8193	0.0175	IL6
	0.9268	0.0022	IL10
*Streptococcus cristatus*	0.8024	0.0211	IL6
*Isosphaera pallida*	0.7365	0.0458	IL8
*Streptococcus mitis*	-0.7350	0.0448	IL8
*Streptococcus pyogenes*	-0.8095	0.0218	IL8
*Methanobrevibacter* sp. AbM4	-0.8095	0.0218	IL8
*Geobacillus* sp. C56-T3	0.8049	0.0216	IL10
*Chlorobium phaeobacteroides*	-0.8796	0.0069	IL10
*Carnobacterium inhiben*	-0.8434	0.0119	Neopterin
*Aromatoleum aromaticum*	0.8095	0.0218	Neopterin
*Bacillus anthracis*	0.7904	0.0251	Neopterin
*Listeria ivanovii*	0.7832	0.0260	TNFa
*Prevotella intermedia*	0.8333	0.0154	TNFa
*Candidatus Pelagibacter ubique*	-0.8932	0.0042	IFN-γ
*Nitratifractor salsuginis*	-0.8193	0.0172	IFN-γ
*Desulfosporosinus orientis*	-0.747	0.0409	CRP
*Streptococcus dysgalactiae*	0.9048	0.0046	CRP
*Methanobacterium* sp. MB1	0.8024	0.0211	CRP
*Streptococcus thermophilus*	0.7904	0.0248	CRP
*Streptococcus lutetiensis*	0.7619	0.0368	CRP
*Streptococcus infantarius*	0.8095	0.0218	CRP
*Streptococcus gallolyticus*	0.8571	0.0107	CRP

Many bacterial species strongly correlate, both positively (grey) and negatively (light blue) with more than one cytokine and more species show significant association with single cytokines only. For this, Spearman r correlation matrix (presuming a non-gaussian distribution) with 95% confidence interval was used. A simple two-tailed Mann-Whitney U test was performed for significance for this correlation matrix.

### Bacterial Association Networks Change With Age

**Figure 6:**
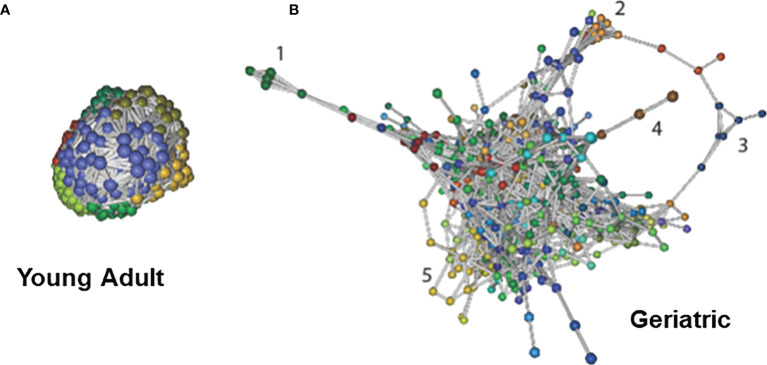
Bacterial association networks and differences with age: Bacterial association networks and differences with age: Markov Clustering Algorithm (MCL) evaluates correlations of vector abundance of bacterial species within each group, thereby generating a cluster map by using correlation scores as distance. Figure shows that microbiome of young adult animals group into a single large super-cluster at microbial homeostasis **(A)**. However, with age, major constraints have been introduced into the network structure with the emergence of satellite clusters and scattering of the super cluster **(B)**. Cluster identities are listed in **Supplementary Figure 4**. Different colors signify different MCL minor clusters within the microbiome. The data used is species-level identified list, and the rendering for MCL includes only those with Pearson’s r > 0.85 and unidentifiable feature-level sequences were not included for this analysis.

Bacterial associations were calculated using Markov Clustering Algorithm (MCL), which evaluates correlations of vector abundance of bacterial species within each group. The rendering for MCL only includes those with pearson’s r > 0.85 and unidentifiable feature-level sequences were not included for this analysis. This calculation results in clustering of species that are most likely to co-occur together in a network, using correlation values as the distance matrix. This approach also allows for each species to participate in multiple clusters, emulating real life participation of individual microbes in different networks and biofilms. As shown in **Figure 6A**, the microbiome of young adult animals exhibited a single large super-cluster at microbial homeostasis. However, in geriatric animals, major constraints have been introduced into the network structure with the emergence of satellite clusters and scattering of the super cluster (**Figure 6B**). In geriatric animals, various newly emerging breakout clusters could be seen with known bacterial composition (Representative clusters numbered, and composition shown in **Supplementary Figure 4**). The investigation of age-induced relative changes in the microbial composition to changes in bacterial associations are depicted in **Supplement Figure 4** showing that bacteria from cluster 1 in **Figure 6B** belong to phyla Archaea, Thermotogae, Proteobacteria and Bacteroidetes. A common property of constituents of cluster 1 was that they were all extreme thermophiles. Similarly, in cluster 2, with the exception of *Lactobacillus casei*, all constituents were known pathobionts (i.e. potential mammalian pathogen). The putative separation/isolation of functionally distinct clusters of microbes to form distinct niches is associated with changes in metabolism and virulence. Hence, in aging, constraints are introduced into the homeostatic microbial networks and bacteria of known traits seemingly cluster together, forming their own niche; this could also result in change in their metabolic profiles.

### Serum Metabolites Show Significant Differences Between Young Adult and Geriatric Animals With Strong Correlation With Altered Microbial Metabolism

**Figure 7:**
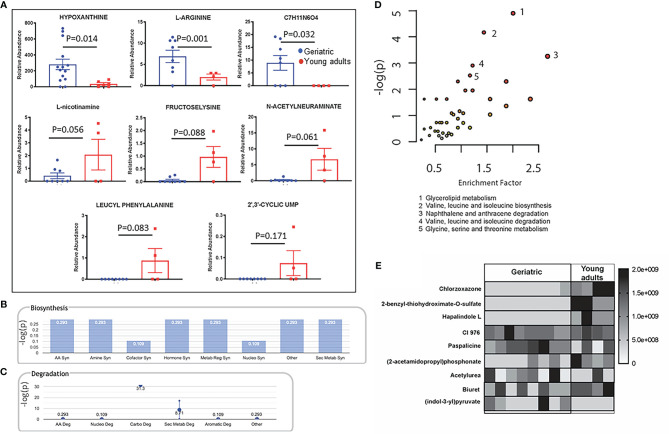
Crosstalk between the bacterial and host metabolic pathways: Mass-spectrometry based Isotope Ratio Outlier Analysis (IROA) showed a few metabolites that were significantly different between the two groups **(A)**. Bar graph showing -log(p) enrichment of the biosynthetic and stress pathways in the geriatric animals upon mapping these metabolites on pathways **(B)**. Enrichment -log(p) of the all degradation pathways with the exception of carbohydrate and secondary metabolite catabolism in geriatric animals **(C)**. LC-MS/MS analysis of the pure microbial fraction and pathway enrichment analysis showed that glycerolipid, amino acid and secondary metabolite metabolism predominated the metabolic landscape of both groups- Red color signifies significantly enriched pathways in geriatric macaques **(D)**. Upon mapping the significantly different metabolites between geriatric and young adult macaques **(E)** to metabolic pathways, a synchrony was observed between microbial and host biosynthetic and degradation pathways. All metabolites used in the analysis were significantly different between young adult and geriatric animals (p < 0.05 in Mann-Whitney U test) with FDR of 0.1 (Benjamini-Hochberg correction).

One of the ways the microbiome can affect the host is through their own metabolism, producing specific metabolites that make their way into the host circulation. Metabolites in the serum of young adult and geriatric animals were analyzed by mass-spectrometry based Isotope Ratio Outlier Analysis (IROA) as previously described (31). Serum from young adult and geriatric animals showed a few metabolites that were significantly different between the two groups (**Figure 7A**). Geriatric animals showed higher levels of hypoxanthine, L-arginine, and an unidentified compound with a chemical composition of C7H11N6O4. Young adult animals showed higher levels of L-nicotinamine, fructoselysine, N-acetylneuraminate, leucyl phenylalanine, and 2’,3’-cyclic uridine monophosphate (UMP). Most of the biosynthetic pathways were found to be upregulated in the geriatric animals (**Figure 7B**). All degradation pathways except for carbohydrate and secondary metabolite catabolism, were higher in geriatric animals (**Figure 7C**).

**Figure 8:**
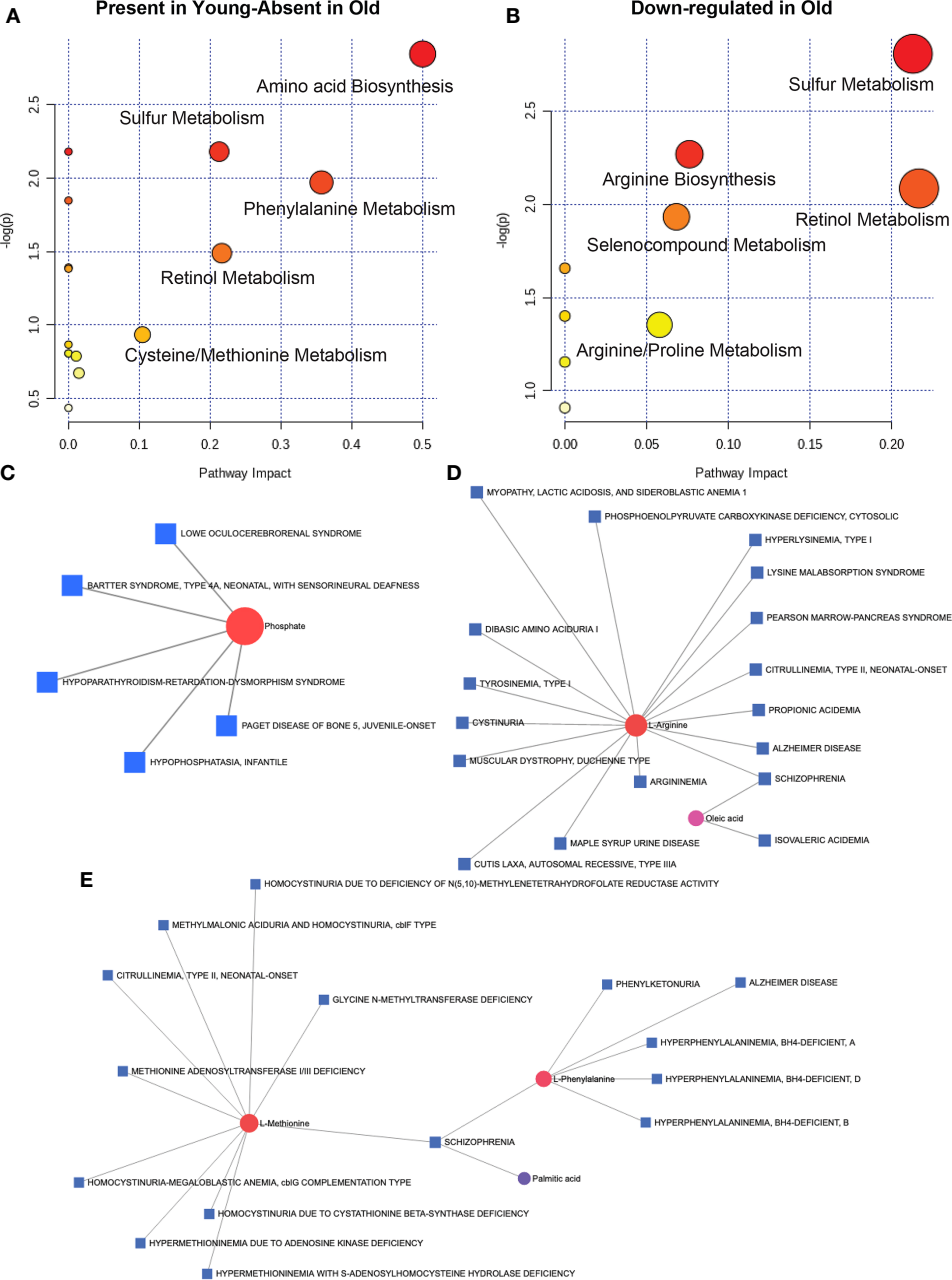
Microbial metabolites and host disease pathways: In the microbe-free fecal compartment, after adjusting for diet-derived metabolites, several metabolites were significantly altered, that were mapped to several pathways in the aged animals. When all significantly changing metabolites in geriatric and young adult animals were mapped for human pathway impact (MetaboAnalyst 4.0),secondary metabolite synthesis, nucleotide synthesis and amino acid synthesis and degradation were still at synchrony with bacterial and host metabolic status for metabolites either absent **(A)** or down-regulated **(B)** in the microbial complement in geriatric animals (also see **Supplementary Table 1**). Metabolite-disease interaction network mapping identified several human diseases, mostly centered around alteration in Phosphate **(C)**, L-Argenine/Oleic acid **(D)** and L-Methionine/L-Phenylalanine **(E)** metabolism. All metabolites used in the analysis were significantly different between young adult and geriatric animals with FDR of 0.1 (Benjamini-Hochberg correction).

To understand the metabolic landscape of the microbial compartment in the gut microbiome of the two age groups, we isolated metabolites from the microbial and microbe-free fecal matter. LC-MS/MS analysis of the pure microbial fraction and pathway enrichment analysis showed that glycerolipid, amino acid and secondary metabolite metabolism predominated in both age groups (**Figure 7D**) but with significantly different metabolites as shown in **Figure 7E**. In the microbe-free fecal compartment, secondary metabolite synthesis, nucleotide synthesis and amino acid degradation remained at synchrony with bacterial and host metabolic status (**Figure 8**). Several metabolites were significantly different between geriatric and young adult RM (**Supplementary Table 2**), which were mapped to various human pathways using KEGG orthologies. *Macaque* chow was used as a control for this experiment and all metabolites present in the chow were excluded from the differential analysis. Significantly different metabolites were mapped on human pathways using MetaboAnalyst 4.0, and significantly impacted pathways were identified for metabolites that were abolished or down-regulated in geriatric macaques (**Figures 8A, B**, respectively). Next, we mapped the altered metabolites to Metabolite-disease interaction networks. Several human diseases were identified, centered around alterations in Phosphate (**Figure 8C**), L-Argenine/Oleic acid (**Figure 8D**) and L-Methionine/L-Phenylalanine (**Figure 8E**) metabolism. Many of these diseases are co-morbidities associated with aging.

## Discussion

Aging is associated with inflammation and changes in immune homeostasis (1, 3, 32, 33), which have been recapitulated in rhesus macaques (16, 34). The relationship of immunologic alterations and inflammatory changes of aging with the composition of the gut microbiome or host metabolic states is not well understood. The geriatric and young adult animals in this study were in a similar environment and on the same diet, underscoring the relationship of the gut microbiome with the host inflammatory and metabolic state that is accentuated with aging. These observations support the concept that microbial manipulations could potentially prove to be an effective tool in mitigating age-associated morbidities resulting from inflammation.

In agreement with the previous reports, our NHP model showed increase in several inflammatory cytokines and soluble markers of immune activation in geriatric compared to young adult animals (16, 35–37). Consistent with increase in neopterin, we also found an increase in IL-6 and IL-8, supporting a generalized state of monocyte/macrophage activation contributing to systemic inflammation in the geriatric animals (38). Significant alterations in circulating immune cell subsets were noted in geriatric compared to young adult RM. Absolute numbers of lymphocytes in geriatric animals were decreased (16) with decreases in frequencies and absolute numbers of total and naive CD4 T cells and increases in the central memory CD4 T cells, suggesting T cell differentiation without production of newer cells, possibly as a result of ongoing inflammation and immune activation. Taken together, the immune profile of the animals recapitulates human aging and supports the use of NHP as a suitable animal model for studying aging and age related complications (16, 35–37).

The gut microbiome is known to change with age (6, 20, 24, 25, 28). In the present study, analysis of the gut microbiome revealed significant differences between the microbiome of young adult and geriatric animals. Differences in the microbiome composition that were observed in the PCA plot were due to class and species-level differences resulting from the taxonomic composition of microbiome with aging in these animals. Unlike humans, dietary influence on microbiome composition was minimal in these animals as they were fed the same diet. Expansion of the investigation of microbiome composition beyond diversity to evaluate clustering patterns, further supported age associated differences due to relative perturbations in bacterial populations. Changes in relative abundance of microbial species imposes constraints on co-occurrence network(s), resulting in induction/expulsion of other species, or complete rearrangement of networks (39). These changes are associated with alteration of biochemical behavior of individual microbes, often resulting in enhanced expression of virulence factors and metabolites that influence host immunity and cause inflammation in advanced age.

We investigated the potential mechanisms linking the microbiome and host immune system and the impact of age on these interactions. Although crosstalk between the microbiome and immune system is implied, underlying mechanisms have been elusive. We first tried to identify associations between systemic markers of inflammation and immune activation with microbes. Our data showed that microbes that were more prevalent in young adult animals were largely negatively correlated with systemic inflammation while microbes prevalent in geriatric animals showed mainly positive correlations with multiple markers of inflammation and immune activation such as CRP and neopterin. Increase in gut permeability with age allows more bacterial products to translocate to the blood and promote inflammation (6, 14, 15, 40). Bacterial products from different microbes could elicit differential inflammatory responses in the blood, and feed back onto the gut to promote gut permeability. In this manner the gut microbes and the host inflammatory response create a positive feedback loop that accumulates with age (41). A link between microbiome, inflammation and immune response to influenza vaccination has been described recently in healthy adults (41). In this study young adult adults were treated with broad-spectrum antibiotics prior and subsequent to seasonal influenza vaccination. Data showed that in subjects with low pre-existing immunity reduction in gut bacterial load was associated with significant impairment in H1N1-specific neutralization and binding IgG1 and IgA responses. Antibiotic treatment resulted in enhanced inflammatory signatures, observed previously in the elderly, and increased dendritic cell activation and divergent metabolic trajectories, with a 1,000-fold reduction in serum secondary bile acids, which was highly correlated with AP-1/NR4A signaling and inflammasome activation (41).

In the current study, bioinformatic and high-throughput methodologies were used to link age-associated microbial changes and corresponding changes in microbial metabolites to age-associated immune and metabolic abnormalities. Determination of serum metabolites was an important component of this study. The microbiome is known to play a major role in metabolism, breaking down food and producing a number of different metabolites that are absorbed by the host. In turn these metabolites can have a number of different effects on the host, including the immune system. Among the significant differences in serum metabolites of young adult and geriatric animals, the compound C7H11N6O4 that could not be identified, has the same molecular formula as urea and might be a part of the Hypoxanthine (increased in geriatric animals) pathway. Hypoxanthine is oxidized to uric acid at the end of purine metabolism, releasing reactive oxygen species (ROS) (42). 2,3-cUMP, which is abolished in the geriatric animals, is a predominant bacterial product, that can be converted to nucleoside-3-phosphate in eukaryotes and its inhibition has be shown to induce mitochondrial stress by modulating Akt and GSK3b activity (43). Fructoselysine is an Amadori product that can be broken down by commensal gut microbes to create butyrate, which in turn has positive effects on the gut mucosa (44, 45). L-nicotinamine is utilized in various pyridine nucleotide cycles involved in NAD or NADP salvage (46) and we found that this metabolite decreases significantly with age. N-acetylneuraminate is the predominant sialic acid used in cells. Leucyl-phenylalanine can be combined with formyl-methionine to make N-formyl-methionyl-leucyl-phenylalanine (FMLP) which acts as a neutrophil chemoattractant and macrophage activator (47). Lastly, l-arginine is a basic amino acid, but is also used as a substrate to generate nitric oxide, which has beneficial effects for the brain and memory (48). All these metabolites have a direct effect on age related co-morbidities and stress pathways.

The commensal bacteria themselves have major differences in lipid metabolism, amino acid metabolism and xenobiotic processing capabilities between geriatric and young adult animals. When we examined the metabolite-level differences in the diet subtracted fraction of the microbial metabolites (the secretome; **Supplementary Table 2**), geriatric RM exhibited diminution of metabolites that protect mammalian cells from toxicity, modulate immune cells (phenyl phosphate, biuret, methyl chloride), afford protection from neurotoxicity (thiobenzamide), modulate sphingolipid and ceramide metabolism (enol-oxaloacetate) and maintain microbial homeostasis. These metabolites are important for efficient vitamin uptake and are also anti-carcinogenic (4-fluoroglutamate (49), (2Z)-2-amino-3-hydroxy-3-(4-hydroxyphenyl) prop-2-enoate (50, 51) and (2S, 3R)-2-[(2-aminophenyl)amino]-3-carboxy-3-oxopropanoate (52)). These observations are based on presence/absence and changes in relative abundance of metabolites from the serum of geriatric and young adult macaques, which may or may not be a consequence of gene and pathway activation. However, downstream participation of these metabolites in various pathways would definitely be consequential to the host. Among the few metabolites that exhibited upregulation in the geriatric animals, O-Phospho-L-Homoserine is a bacterial quorum-sensing molecule, which is known to participate in various host metabolic pathways (**Supplementary Figure 5A**). In addition, excess amount of this molecule is known to modulate macrophage activation and cytokine synthesis, in favor of gram-negative bacterial infection in host (53). Another compound, upregulated in geriatric animals, 3,4-Dichloroaniline is a bacterial/fungal degradation product of toxic dietary compounds (e.g. linuron, propanil etc.; **Supplementary Figure 5B**) and known to induce fatty liver disease and cytotoxicity in zebrafish and rats (54, 55).

Our study limited by the small sample size, gender difference between young adult and geriatric animals and distant relatedness of the animals. The use of primarily female geriatric animals was due to the fact that these animals were retired from breeding (study naïve) and at >18y old, were truly aged and males of this age are not available as generally they have been enrolled in other studies. Another limitation is that the fecal microbiome can vary within an individual, and a single time-point may be stochastic, in very small group sizes. Despite the modest number of animals per group this systematic analysis of microbiome and metabolites identified a significant age associated alteration in microbiome profiles reflective of alterations at the individual animal level.

In summary, the rhesus macaque appears to reliably portray age-associated changes in the microbiome and immunity. In the context of aging, as demonstrated in **Figures 5**, **7** and **8**, microbial changes can affect energy homeostasis, proliferation and immune homeostasis in the host and microbial metabolites play a major role in fostering these changes. These data also imply that microbial manipulations could potentially prove to be an effective tool in mitigating age-associated morbidities resulting from inflammation in the aging population. The correlation of dysbiotic microbiome with inflammation and its relationship with microbial metabolism in aging, point to future directions for investigating how age-associated changes in the microbiome are linked to changes in age-associated morbidities. Additionally, understanding how the microbiome and the immune system affect each other could open the door to better therapeutic interventions for immune and metabolic diseases in the aging population.

## Data Availability Statement

Sequence files for the samples in this article have been submitted to ArrayExpress with the accession number E-MTAB-10160. Most of the analysis was performed on PartekFlow platform (BowTie, STAR, KRAKEN), which is proprietary. The rest are publicly available (MetAmos, Explicet, Graphia).

## Ethics Statement

All animal experiments were conducted following guidelines established by the Animal Welfare Act and the NIH for housing and care of laboratory animals and performed in accordance with institutional regulations after review and approval by the Institutional Animal Care and Usage Committees (IACUC, # 2016-8798-055) of the University of Louisiana at Lafayette.

## Author Contributions

SuP, SB, and SaP provided intellectual input and contributed to the experimental design. SuP, performed immunology experiments, data analysis and manuscript writing. SB, study conception and design, data analysis, bioinformatics analysis, and manuscript writing. SaP, study conception and design, manuscript writing, and final draft revision. RM, microbiome and metabolome experiments. KR, assisted with immunology experiments. DK, immunology data analysis. RP, study design and manuscript editing. TS, animal studies and sample collection. FV, study design, sample collection, and manuscript editing. All authors contributed to the article and approved the submitted version.

## Funding

This study has been funded by grants awarded to SaP and FV (R01AI123048), to SaP and SuP (R01AG068110) Laboratory Sciences Core of the Miami Center for AIDS Research (CFAR) (P30AI073961) and Miami CFAR pilot and Institutional research support to SB.

## Author Disclaimer

This work was prepared while SB was employed at University of Miami. The opinions expressed in this article are the author’s own and do not reflect the view of the National Institutes of Health, the Department of Health and Human Services, or the United States government.

## Conflict of Interest

The authors declare that the research was conducted in the absence of any commercial or financial relationships that could be construed as a potential conflict of interest.

## Publisher’s Note

All claims expressed in this article are solely those of the authors and do not necessarily represent those of their affiliated organizations, or those of the publisher, the editors and the reviewers. Any product that may be evaluated in this article, or claim that may be made by its manufacturer, is not guaranteed or endorsed by the publisher.
