# Supplementary material for: Age Associated Microbiome and Microbial Metabolites Modulation and Its Association With Systemic Inflammation in a Rhesus Macaque Model
Source: Front Immunol. 2021 Oct 19;12:748397. doi: 10.3389/fimmu.2021.748397 (PMC8560971; doi:10.3389/fimmu.2021.748397)
Supplement: Supplementary file 1 [file DataSheet_1.docx]

**Supplement Information**

**Supplementary Figures**

**
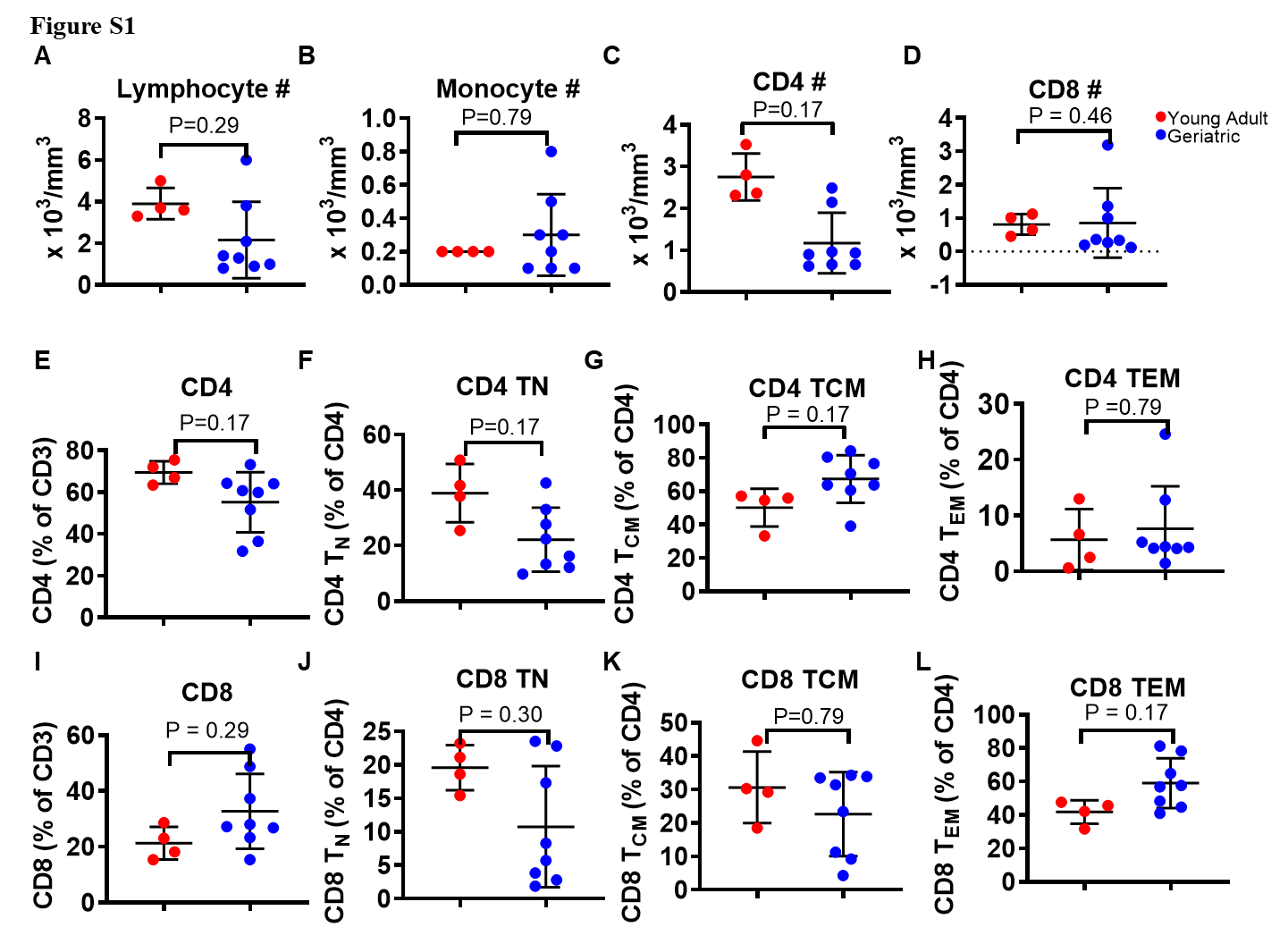
**

**Figure S1: Absolute numbers and frequencies of CD4 and CD8 T cells in young adult and geriatric Rhesus Macaques:** Blood counts was done to determine absolute counts of and flow cytometry for frequencies of CD4 and CD8 T cells from PBMC. Absolute numbers of lymphocytes (A), monocytes (B), CD4 (C), and CD8 (D), T cells in young adult and geriatric Rhesus Macaques. Frequencies of CD4 (E), naïve CD4 (T_N_) (F), central memory (T_CM_) (G), and effector memory (T_EM_) (H), CD8 T cells (I), naïve CD8 (J), TCM CD8 (K), and TEM CD8 (L), T cells. Statistical analysis performed by Mann-Whitney U test with Benjamini-Hochberg correction for multiple comparisons to calculate adjusted p-values (q value). Line and whiskers indicate the mean ± standard deviation. Q values are exact and a q value <0.05 was considered significant.

**
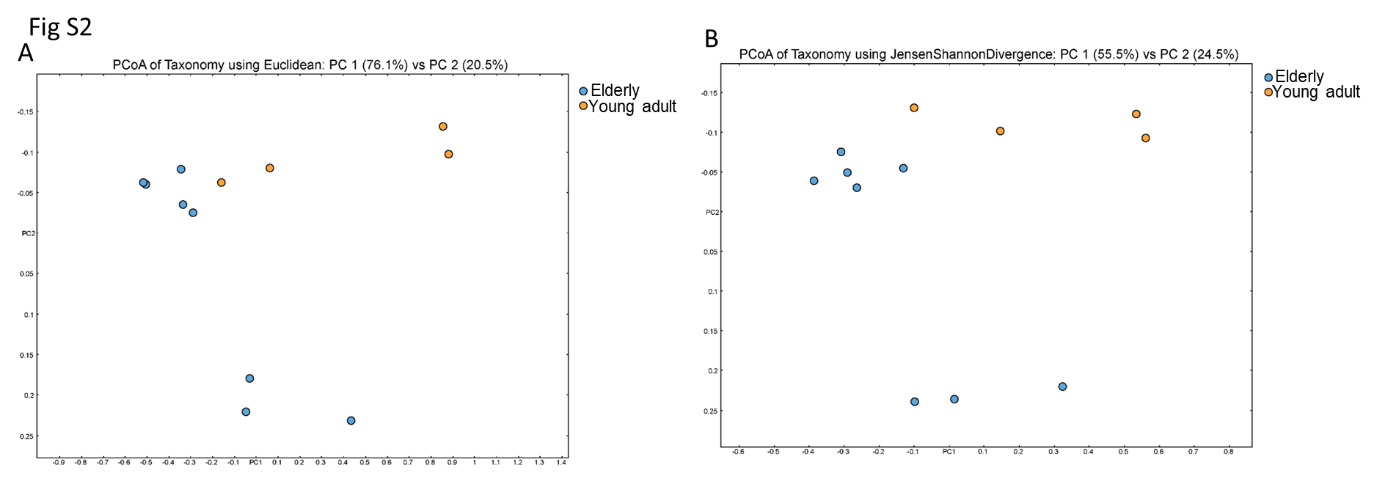
**

**
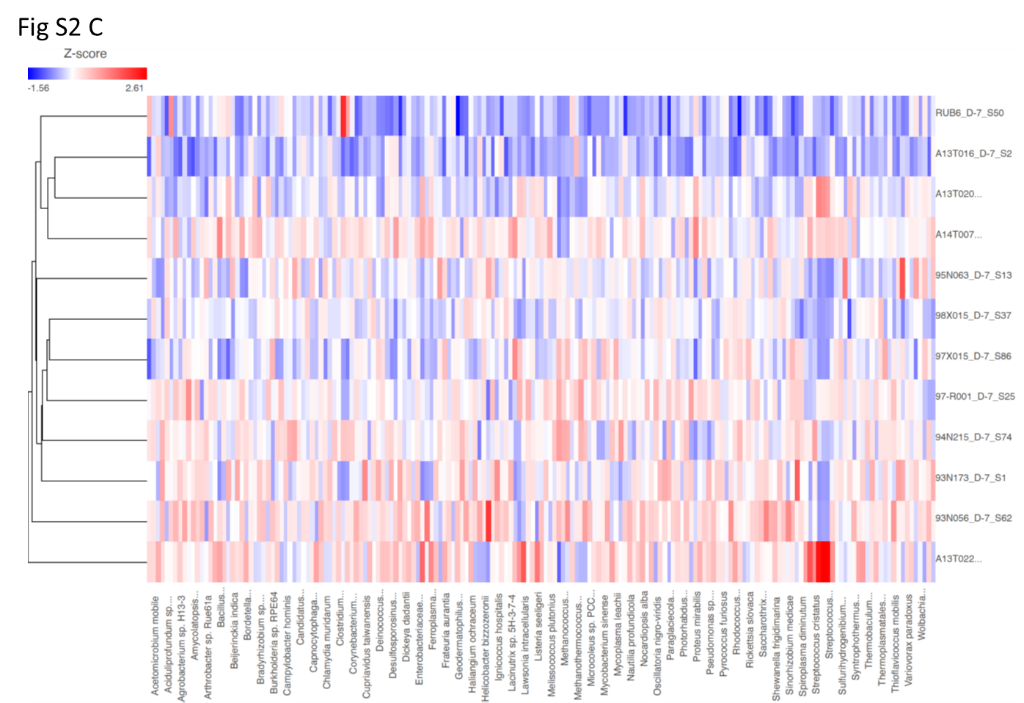
**

**Figure S2**: Euclidean (A) and Jansen-Shannon (B) PCA matrices showing microbiome composition differences between young and old animals. Unsupervised hierarchical clustering plot representing microbiome composition of each animal (C).

**Supplementary Figure 3:**

**
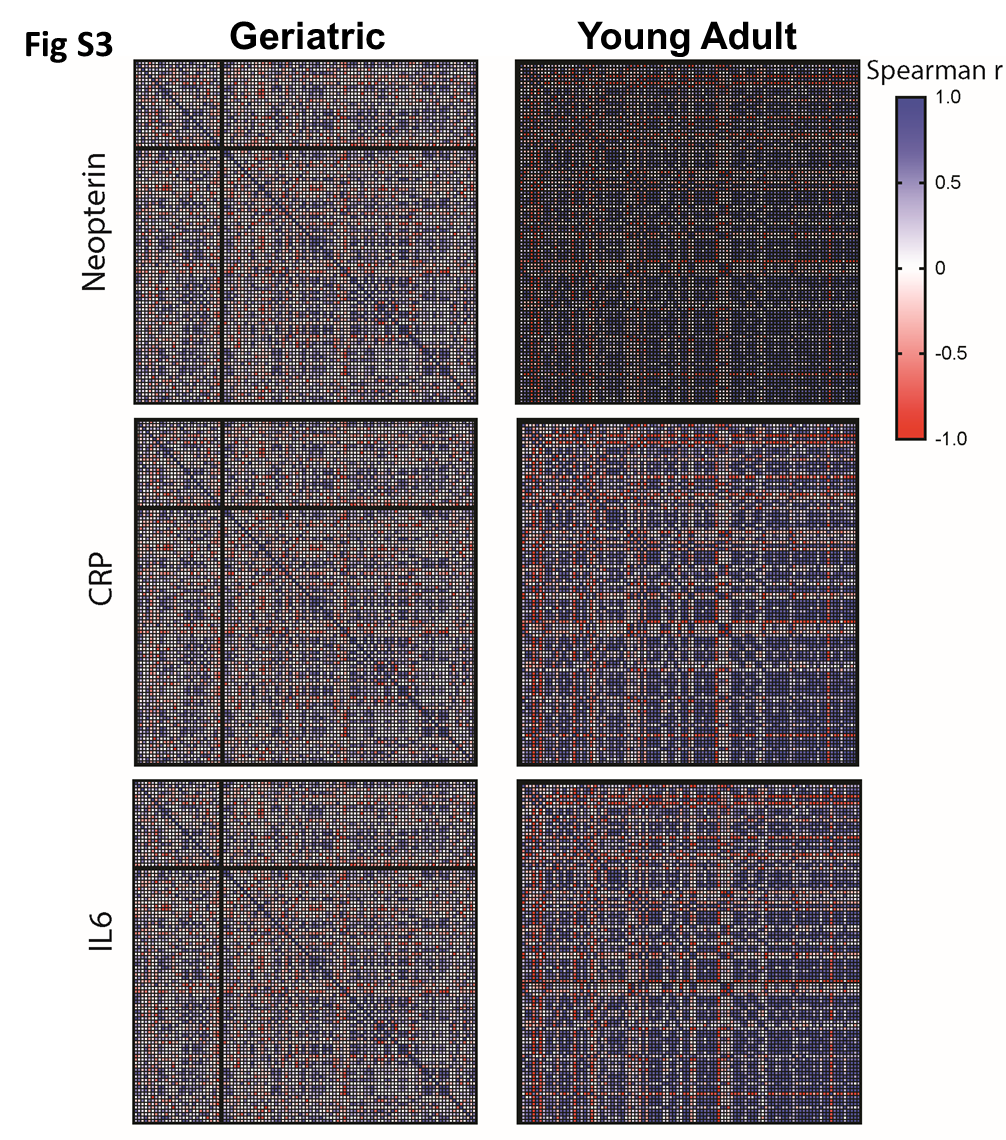
**

**Supplementary Figure 3**: Correlation matrices for CRP, IL6 and Neopterin. Correlation modeling of 100 significantly changing microbial species between the two groups and the cytokine levels in young and old macaques. For this, Spearman r correlation matrix (presuming a non-gaussian distribution) with 95% confidence interval was used. A simple two-tailed Mann-Whitney U test was performed for significance for this correlation matrix.

**
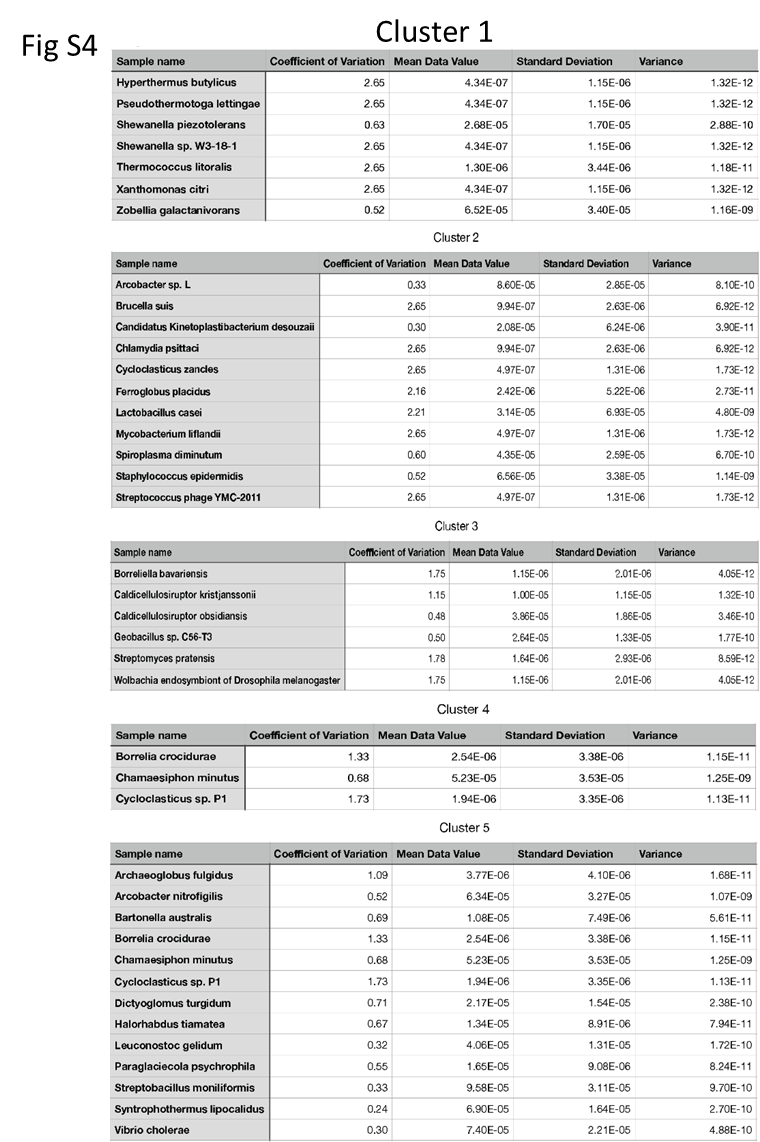
**

**Supplementary Figure 4:** Species composition of individual microbial clusters identified for old animals in main Figure 6. This list was generated from the Graphia’s analysis of MCL data. The Software converts all relative abundance values to ‘percentage of total’ and labels it as ‘mean data value’. Threshold prevalence, pearson’s r and granularity were used as filters.


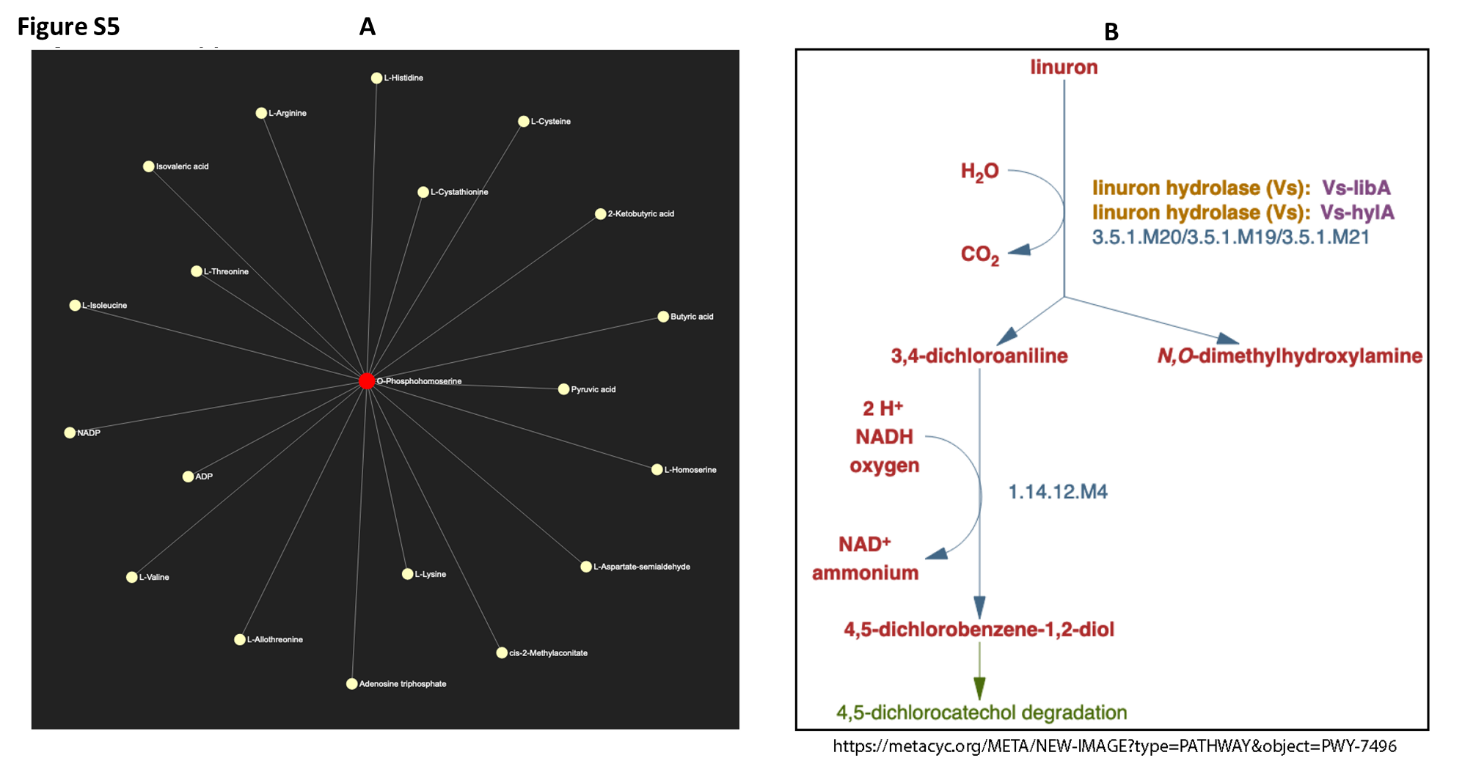


**Supplementary Figure 5:** Bacterial metabolites enhanced in aging Macaques: A), Interaction of O-Phosphohomoserine with other metabolites and pathways within microbiome and with host. B), Pathway association of 3,4-Dichloroaniline in bacteria and fungi.
