# Supplementary material for: Age Associated Microbiome and Microbial Metabolites Modulation and Its Association With Systemic Inflammation in a Rhesus Macaque Model
Source: Front Immunol. 2021 Oct 19;12:748397. doi: 10.3389/fimmu.2021.748397 (PMC8560971; doi:10.3389/fimmu.2021.748397)
Supplement: Supplementary file 2 [file Table_1.pdf]

Supplement Table 1: Characteristics of the animals used in the study

Animal ID	Age Group	Birthdate	Age at Study Entry	Gender	Housing	Relatedness	Date of sampling	Body weights at the time of sampling (kgs)	Body condition score	Last antibiotic treatment	Last NSAIDs treatment	Health status
**93N173**	Old	6/20/1993	24	Male	single	cousin to 95N063, uncle to 97X005, 93N056	**7/17/2017**	**10.65**	**4.5**	**N/A**	**27-Apr-14**	**obese**
**95N063**	Old	5/7/1995	22	Female	single	cousin to 93N173, 93N056	**7/17/2017**	**6.75**	**N/A**	**10-Jan-08**	**N/A**	**healthy**
**97X005**	Old	4/19/1997	20	Female	single	nephew to 93N173, cousin to 98X015	**7/17/2017**	**6.95**	**3**	**7-Feb-11**	**4-Mar-16**	**healthy**
**98X015**	Old	7/10/1998	19	Female	single	cousin to 97X005	**7/17/2017**	**10.6**	**5**	**3-May-04**	**14-Feb-15**	**obese**
**93N056**	Old	4/27/1993	24	Female	single	nephew to 93N173, cousin to 95N063	**7/19/2017**	**8.40**	**N/A**	**14-May-03**	**N/A**	**healthy**
**94N215**	Old	11/12/1994	23	Female	single		**7/19/2017**	**9.25**	**N/A**	**9-Mar-16**	**1-May-12**	**healthy**
**97-R001**	Old	1/1/1994	23	Female	single		**7/19/2017**	**5.35**	**2.5**	**12-Mar-13**	**21-Aug-16**	**healthy**
**RUB6**	Old	4/23/1997	20	Female	single	dam to A13T020	**7/19/2017**	**7.30**	**3.5**	**N/A**	**N/A**	**healthy**
**A13T016**	Young	6/5/2013	4	Male	single	nephew to A14T007	**7/19/2017**	**5.35**	**2.5**	**N/A**	**N/A**	**healthy**
**A13T020**	Young	6/23/2013	4	Male	single	offspring of RUB6	**7/19/2017**	**5.55**	**2.5**	**17-Mar-14**	**N/A**	**healthy**
**A13T022**	Young	7/20/2013	4	Male	single		**7/19/2017**	**4.95**	**2.5**	**N/A**	**N/A**	**healthy**
**A14T007**	Young	5/27/2014	3	Male	single	uncle of A13T016	**7/19/2017**	**4.80**	**2.5**	**N/A**	**N/A**	**healthy**
