# Supplementary material for: Age Associated Microbiome and Microbial Metabolites Modulation and Its Association With Systemic Inflammation in a Rhesus Macaque Model
Source: Front Immunol. 2021 Oct 19;12:748397. doi: 10.3389/fimmu.2021.748397 (PMC8560971; doi:10.3389/fimmu.2021.748397)
Supplement: Supplementary file 3 [file Table_2.pdf]

Present in Young adults, Absent in Geriatric	Mean (young) AUC	SD	P-value (Benjamini-Hochberg corrected)
L-Methionine	3.30E+15	1.80E+13	0.00139999
4-aminobutanal	1.88E+16	3.60E+05	0.02732292
paspalicine	1.47E+15	4.71E+09	2.5888E-09
Dimethyl trisulfide	2.11E+08	9.70E+03	0.02732789
(S)-Ureidoglycolate	3.16E+13	9.98E+09	0.02733064
enol-oxaloacetate	1.96E+16	3.88E+07	0.02733975
phenylphosphonate	1.00E+16	2.23E+06	0.02734137
Crotanecine	8.88E+12	1.12E+04	0.02735396
Proxan	1.22E+16	2.70E+05	0.02736192
Dimethylsulfonioacetate	9.90E+11	7.50E+06	0.02736272
N-(Acetyloxy)benzenamine	2.70E+14	3.50E+09	0.02737433
2-Methylthiobenzothiazole	6.83E+15	2.80E+05	0.0274043
N-Methylethanolamine phosphate	2.09E+10	1.10E+05	0.02743136
L-Phenylalanine	8.99E+16	1.12E+10	0.02744797
Sodium fluoroacetate	1.19E+12	9.91E+04	0.02745065
3-(2-Hydroxyphenyl)propanoate	2.20E+09	8.60E+03	0.02745716
Benzyl thiocyanate	2.55E+14	1.19E+11	0.02753093
2-Chloro-4-hydroxy-6-amino-1,3,5-triazine	1.43E+13	9.80E+12	0.02754867
2,3-Dimethylmaleate	5.71E+14	2.38E+12	0.0281087
3-Methyl-2-oxobutanoic acid	6.02E+14	8.77E+04	0.02813047
(2Z)-2-amino-3-hydroxy-3-(4-hydroxyphenyl)prop-2-enoate	7.45E+15	1.11E+11	0.028274
2-Aminobenzenesulfonate	3.30E+15	2.01E+13	0.02827475
4-chlorocatechol	1.82E+16	9.99E+09	0.02843428
Phosphotyrosine	7.10E+15	7.20E+11	0.02848907
Diphenidol	9.00E+15	7.27E+11	0.02857142
(S)-Malate	8.88E+13	2.29E+08	0.02858907
Sodium diethyldithiocarbamate	1.96E+15	9.22E+12	0.02971486
2-Pyridyl hydroxymethane sulfonic acid	2.04E+16	4.77E+13	0.03040367
Thiomorpholine 3-carboxylate	1.81E+17	1.77E+12	0.03084678
Bronopol	6.08E+15	6.43E+11	0.03140168
Benzyl alcohol	1.12E+15	8.73E+10	0.03303162
acivicin	4.01E+17	1.15E+09	0.03321789
4-amino-3-methoxybenzoate	3.54E+16	1.21E+14	0.04084084
4-Hydroxy-2-butynal	3.01E+15	3.32E+12	0.04803409
Sulfate	7.91E+15	6.31E+12	0.01096798
4-fluoroglutamate	8.72E+13	1.54E+11	0.0273234
4-Bromophenol	7.99E+13	8.77E+08	0.02733405
Cyclic 2,3-bisphospho-D-glycerate	1.30E+13	9.97E+09	0.02734942
GA	1.19E+16	1.22E+11	0.02735024
Phenelzine sulfate	5.12E+12	7.75E+07	0.02741934
5-Hydroxyindoleacetylglycine	9.46E+15	2.75E+11	0.02758884
brassicicene E	1.60E+13	3.34E+11	0.02759978

Hexadecanoic acid	8.17E+14	3.34E+12	0.02762509
Sethoxydim	2.44E+16	2.14E+10	0.02809963
Cyclic 2,3-bisphospho-D-glycerate	1.81E+14	4.43E+10	0.02987916
Esprocarb	7.78E+14	9.88E+12	0.04034487
Sulfate	8.22E+12	2.94E+10	0.04179767
3,4-Dichloroaniline	8.23E+15	7.75E+09	0.04191133
Sethoxydim	5.29E+14	4.47E+12	0.04255662
Phenelzine sulfate	2.41E+13	2.12E+11	0.04496404
5-Hydroxyindoleacetylglycine	2.00E+13	3.13E+10	0.04879065
Ergonovine	6.43E+14	8.84E+12	0.04946701

**Down-regulated in Geriatric**	Mean (young) AUC	SD (young)	Mean (old) AUC	SD (old)	**P-value (Benjamini-Hochberg corrected)**
2'-aminobiphenyl-2,3-diol	3.16E+15	8.82E+10	2.25E+04	7.00E+02	0.00431054
2-Hydroxypyridine	3.66E+14	1.87E+12	7.22E+10	2.79E+05	0.0003155
2,3,5-Trihydroxytoluene	2.88E+16	1.01E+13	5.39E+09	1.59E+07	0.00112588
Maleamate	7.69E+16	1.12E+15	4.22E+08	2.25E+06	0.04047555
DL-norvaline	2.61E+16	8.97E+13	5.12E+09	4.10E+07	0.04561044
L-Selenocysteine	4.07E+15	2.66E+12	8.51E+11	2.11E+08	0.0433131
Retinoate	1.18E+16	1.19E+09	7.55E+05	9.00E+03	0.04065873
Halacrinate	2.99E+13	5.54E+11	6.64E+08	1.21E+06	0.04231169
(9Z)-Octadecenoic acid	1.97E+15	1.12E+14	2.97E+13	4.54E+10	0.04881578
5-Hydroxyindoleacetylglycine	1.68E+15	9.92E+13	8.77E+09	1.01E+09	0.00701656
Cyclic 2,3-bisphospho-D-glycerate	4.06E+14	2.27E+13	5.54E+12	1.66E+12	0.04134309
glorin	3.56E+13	9.77E+12	1.59E+10	2.58E+09	0.04465609
N-Methylindolo[3,2-b]-5alpha-cholest-2-ene	1.49E+14	7.99E+12	8.89E+09	3.94E+08	0.02359062
Jatrophone	6.84E+15	8.22E+13	6.87E+11	9.44E+10	0.03969174
7,8-Dihydroneopterin	1.02E+16	3.57E+12	2.99E+05	1.49E+04	0.01869177
4-Chlorophenylacetonitrile	3.51E+13	8.77E+11	2.04E+10	4.64E+09	0.04069298
Halacrinate	1.58E+17	9.88E+16	1.84E+04	8.79E+03	0.04467077
Cyclic 2,3-bisphospho-D-glycerate	8.89E+12	7.46E+11	6.73E+09	7.67E+08	0.01290866
Lichenin	4.82E+14	8.02E+10	2.93E+12	9.91E+11	0.0481721
AA861	2.31E+15	1.97E+12	7.79E+07	2.79E+03	0.0097712
Orthophosphate	7.59E+14	1.22E+13	6.54E+05	1.18E+04	0.02772643
L-Arginine	2.75E+16	7.76E+12	9.88E+12	3.28E+08	0.01925691
5-Hydroxyindoleacetylglycine	4.79E+13	4.24E+10	3.86E+11	2.42E+08	0.02663235
(2S,3R)-2-[(2-aminophenyl)amino]-3-carboxy-3-oxopropanoate	9.82E+15	4.32E+14	6.71E+09	8.98E+08	0.0322514

**Upregulated in Geriatric**	Mean (young) AUC	SD (young)	Mean (old) AUC	SD (old)	**P-value (Benjamini-Hochberg corrected)**
3,4-Dichloroaniline	7.79E+14	8.66E+11	2.11E+17	1.01E+14	0.00330997
O-Phospho-L-homoserine	5.87E+11	2.95E+08	7.74E+16	2.39E+15	0.04016044
